# Arthroscopically assisted reduction and internal fixation of a femoral anterior cruciate ligament osteochondral avulsion fracture in an 11-year-old girl: A case report

**DOI:** 10.1097/MD.0000000000030321

**Published:** 2022-09-02

**Authors:** Zhongren Zheng, Lei Wang, Ke Tian, Xiaowei Zhao, Longfei Ma

**Affiliations:** a School of Clinical Medicine, Jining Medical University, Jining, People’s Republic of China; b Department of Orthopedics, The Affiliated Hospital of Jining Medical University, Jining, People’s Republic of China.

**Keywords:** anterior cruciate ligament, arthroscopic assistance, case report, femoral avulsion fracture, knee injuries

## Abstract

The anterior cruciate ligament (ACL) is relatively rarely injured in children and adolescents, accounting for just 0.5% of all ACL rips that occur. Avulsion fractures are more common in youngsters because their ligamentous structures are known to be stronger than their physical insertion sites. Tibial eminences have been reported to be the sites of most ACL avulsions, and both cartilaginous and osteochondral avulsion fractures have been observed, whereas the latter occurs more commonly. On the other hand, femoral osteochondral avulsion fractures of the ACL in children are uncommon, as only a few studies describe their occurrence in immature patients.

**Patient concerns:**

In this case report, we present an 11-year-old girl who suffered an ACL femoral attachment avulsion fracture after pivoting her knee during riding. A comprehensive formal evaluation of the knee was impractical due to the persistence of pain and tight haemarthrosis.

**Diagnoses:**

Femoral anterior cruciate ligament osteochondral avulsion fracture.

**Interventions:**

We used Two No.2Ethibond sutures to pick up the osteochondral fragment and passed across the lateral femoral condyle to come out laterally and fixed with a tie proximally, and we recommended the patient perform reasonable functional exercises postoperatively.

**Outcomes:**

The patient had no pain, instability, or activity limitations after 24 months of surgery. Physical examination of the patient revealed full and symmetric ROM, and normal Lachman and pivot shift test performance.

**Lessons:**

ACL avulsion fractures can be accurately treated with arthroscopic reduction and sutures via an inside-out technique, which can reduce the risk of persistent ligamentous laxity and reduce open surgery-related morbidity.

## 1. Introduction

Children and adolescents suffer from fewer ACL injuries than adults, accounting for only 0.5% of all ACL tears.^[1]^ Furthermore, as the ligamentous structures are stronger than their osseous attachments among children and adolescents, bone avulsions are more commonly seen than intrasubstance ligament injuries; characteristically from the ligament’s tibial connection, resulting in a tibial spine avulsion fracture.^[2–4]^ On the other hand, avulsions of the ACL originating from the femoral are uncommon, with only thirteen cases reported in the literature.^[5–17]^ In this study, we describe the case of an 11-year-old girl who suffered an ACL femoral ending osteochondral avulsion injury which was treated arthroscopically with sutures applied from an inside-out pattern.

## 2. Case report

**Figure 1.**
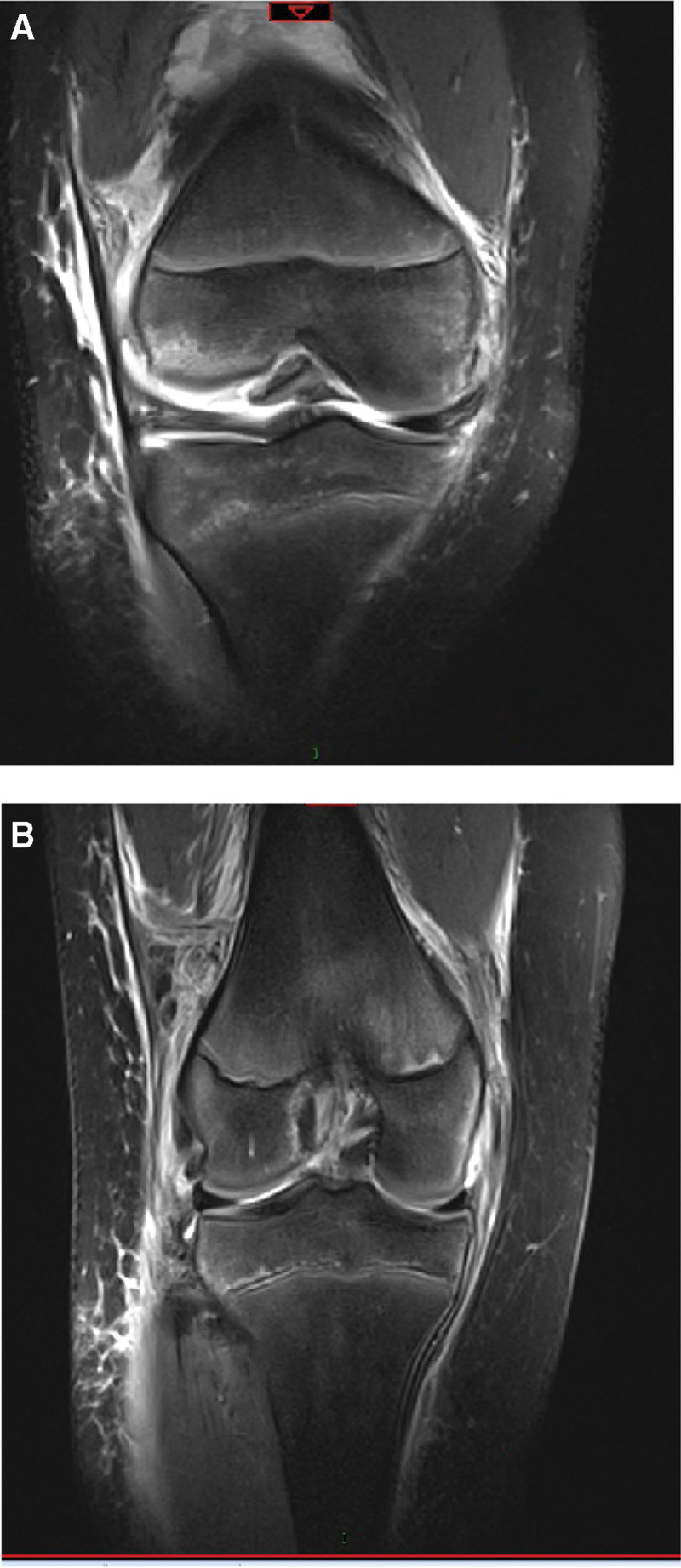
(A, B) MRI scan image of the right knee demonstrated a large lateral femoral condyle contusion with an ACL rupture near its femoral origin.

**Figure 2.**
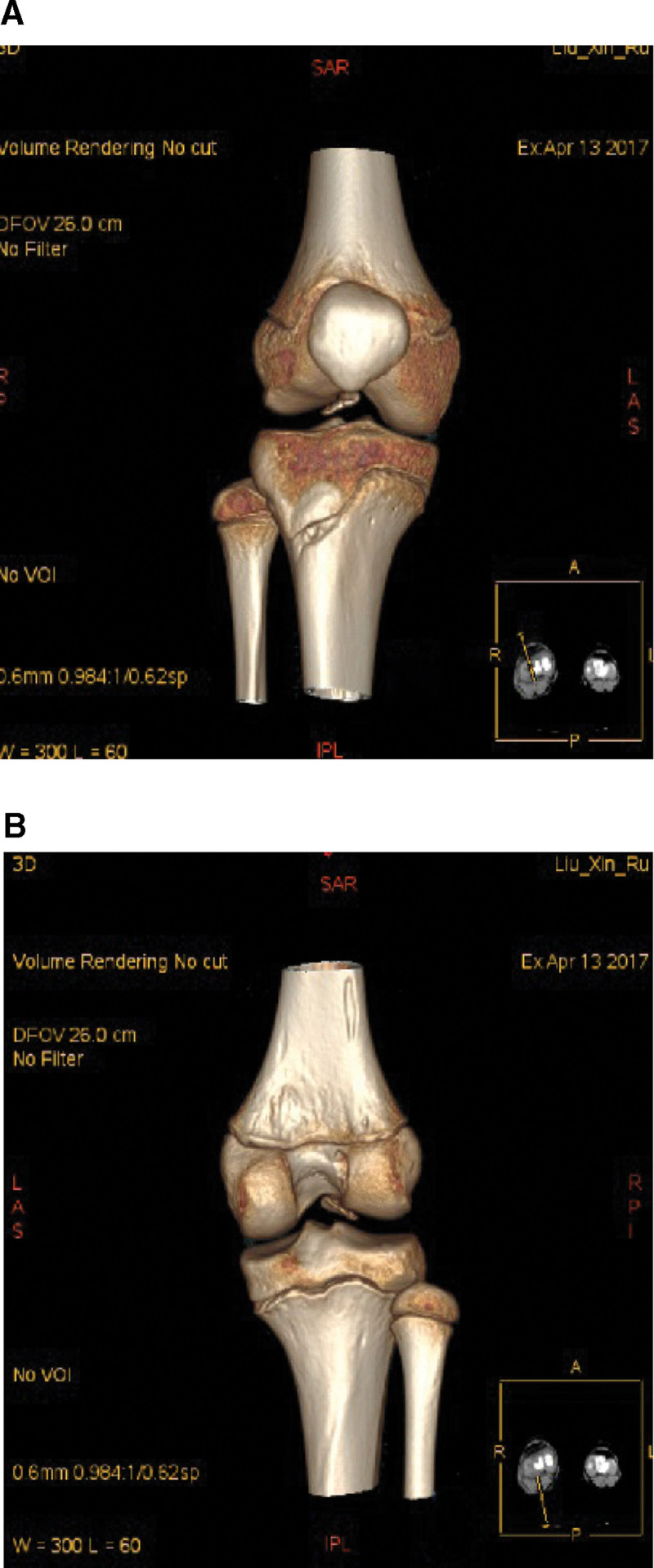
(A, B) CT scan image clearly shows that the lateral femoral condyle had been fractured posteromedially within the intercondylar notch at the proximal ACL junction.

A girl (11 years old) suffered from a curving injury in her right knee while freeing. She quickly had a torment and uneasiness in her knee, as a result, she could not broaden her joint. A full accurate assessment of her knee was impractical because of torment and strained haemarthrosis. The magnetic resonance imaging (MRI) scans of the right knee showed an ACL rupture at its femoral origin and a significant lateral femoral condyle contusion (Fig. 1). Computer tomography (CT) scans were conducted to better understand the injury. According to the scan images, there was a comminuted fracture of the posteromedial portion of the lateral femoral condyle near the proximal ACL attachment within the intercondylar notch (Fig. 2). Based on these findings, we decided to fix the fracture fragment surgically.

**Figure 3.**
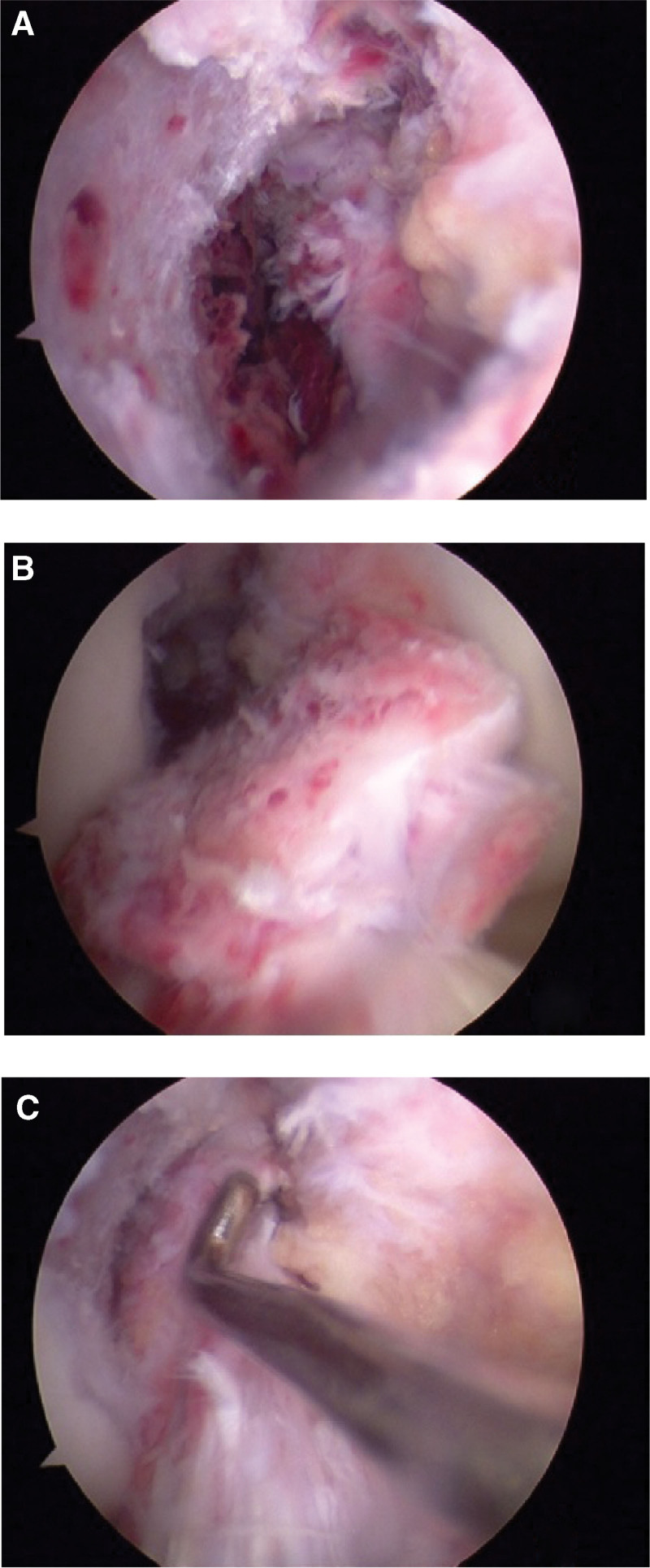
(A) In a diagnostic arthroscopy, the ACL was found to be avulsed from the femur; (B) multiple bone fragments were found to be adhering to the femoral section of the ligament; (C) the intrasubstance of the ACL remains intact.

An avulsion of the ACL at the femoral end was confirmed by diagnostic arthroscopy, with several bone fragments still connected to the femoral portion of the ligament and no intrasubstance damage was observed (Fig. 3).

**Figure 4.**
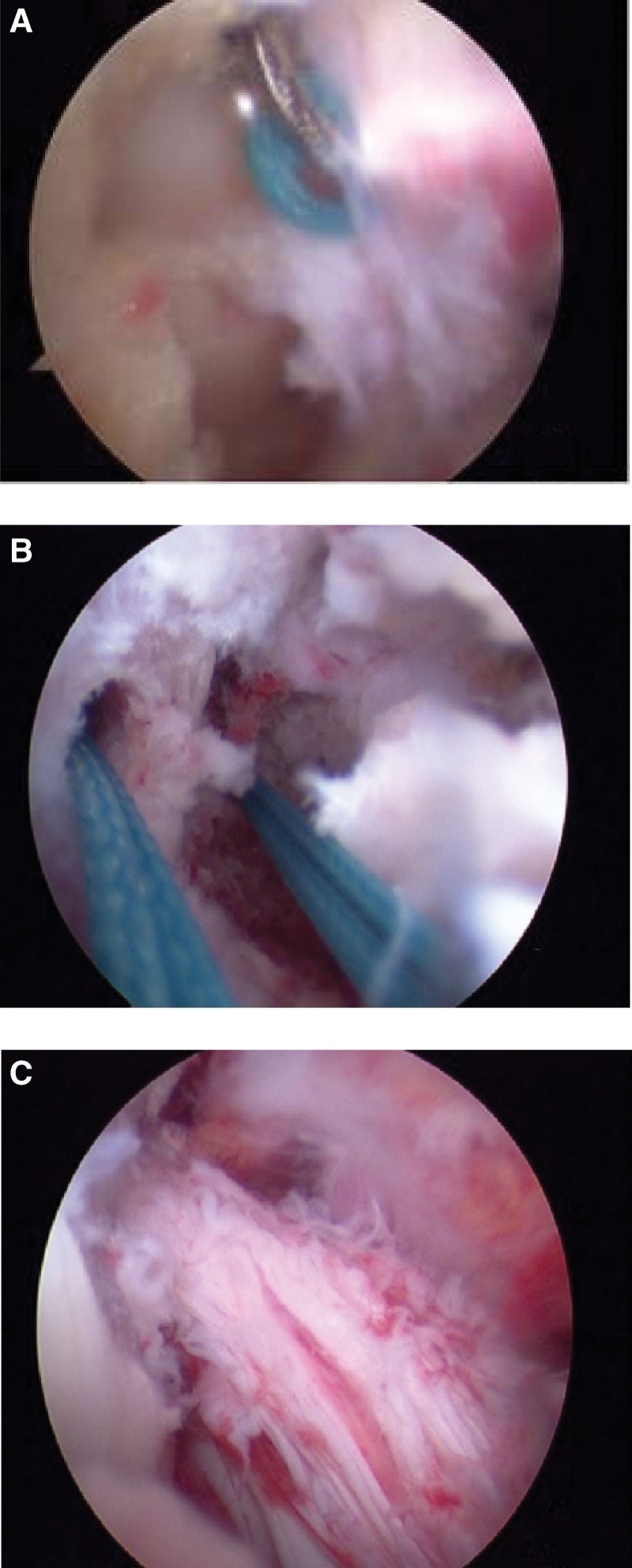
(A) The sutures were inserted across the ACL’s femoral end. (B) The sutures were inserted across the lateral femoral condyle; (C) Avulsed fragments were stitched back to their normal anatomical positions.

Under arthroscopic visualization, two No. 2Ethibond sutures were sutured across the femoral end of the ACL, followed by two 2.4 mm guiding pins with sutures drilled inside-out into the middle of the osseous defect of the lateral femoral condyle. By passing the sutures over the lateral condyles of the femur and coming out on the other side, we pulled back the fragment into its natural anatomical position and tie it proximally. Following the surgical repair, the ACL tension was clinically normal and the ACL was restored (Fig. 4).

**Figure 5.**
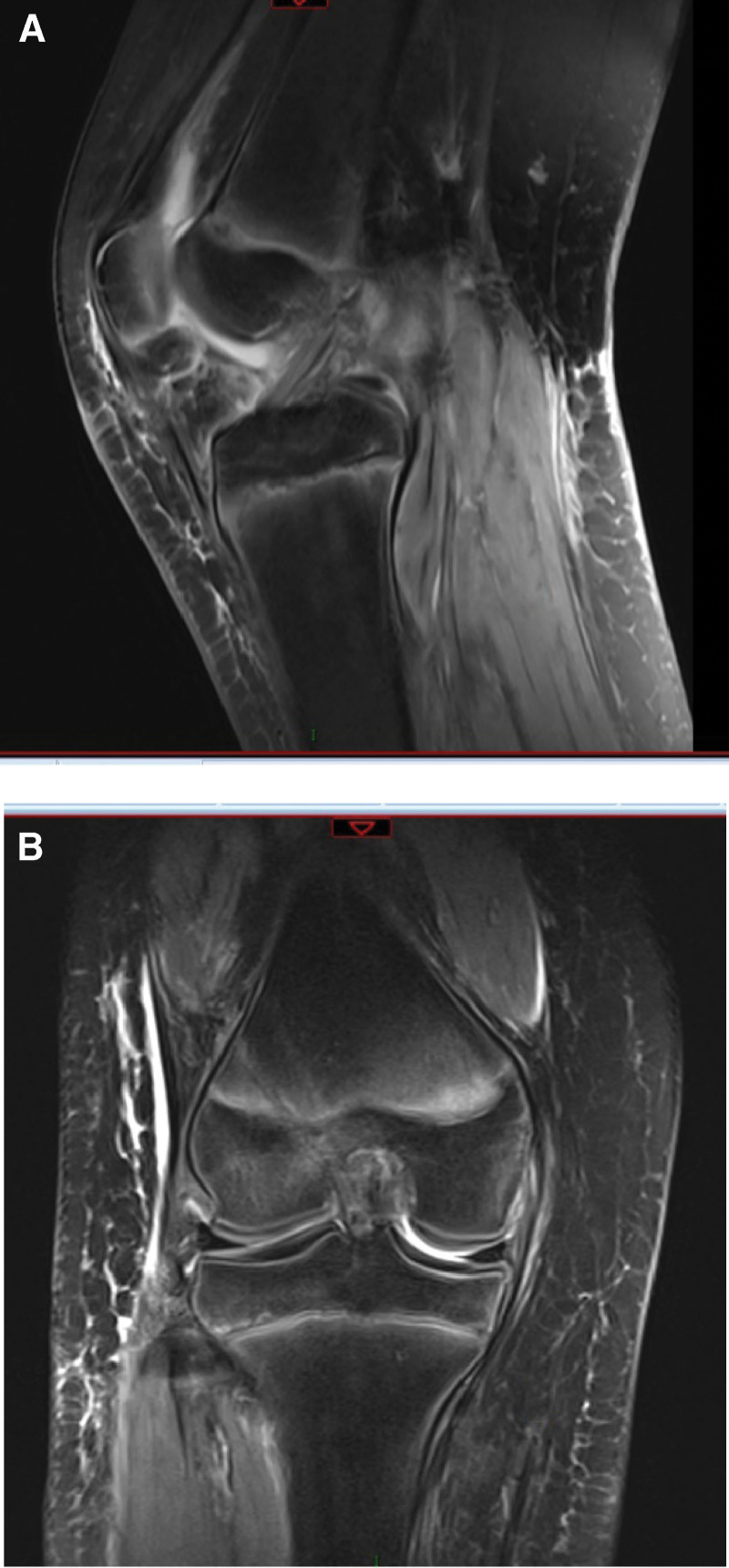
(A) An MRI scan image of the Sagittal side revealed good morphology of the ACL following the definitive fixation; (B) An MRI scan image of the Coronal side showed good morphology after the definitive fixation.

From the day following surgery, quadriceps setting and straight leg raising exercises were recommended. Additionally, they were recommended to wear a knee immobilizer in a fully extended position with the foot flat for the first 4 weeks postsurgery. Crutches were allowed for partial weight-bearing. An MRI revealed that the ACL morphology was good 4 weeks after the surgical fixation (Fig. 5). Within 2 weeks, the patient was recommended to wear a hinged knee brace that allowed her up to sixty degrees of flexion. Six weeks later, the patient began formal rehabilitation. After 3 months, the patient was able to walk without the brace. Approximately, 24 months postsurgery, she experienced no sign of pain, instability, or limitations of activity. The physical examination revealed a full and symmetric range of motion (ROM), as well as normal Lachman and pivot shift tests, which evaluated the effectiveness of the procedure.

## 3. Discussion

ACL tears in children and teenagers are rare, making up just approximately 0.5% of all ACL tears.^[1]^ There are very few cases of anterior cruciate ligament avulsions of the lateral femoral condyle. The majority of ACL injuries in children are caused by tibial eminence osteochondral avulsion fractures. ACL avulsions at the femoral end are epidemiologically prevalent and are categorized into 2 types: cartilaginous avulsions^[6,9]^ and osteochondral avulsion injuries,^[5,7,8,10–17]^ the latter of which occurred in our patient whose case we presented in this study.

Increasing evidence indicates that ACL ruptures among children are more common than previously thought and that poor clinical outcomes are most likely related to conservative management of the case.^[8,18]^ ACL avulsion fractures of the femoral condyle can be repaired openly using a medial parapatellar incision and pullout sutures through drill holes, as reported by many authors.^[7,14]^ After that, an increasing number of specialists performed arthroscopic repairs, resulting in excellent functional results and cosmetic results as a long surgical scar is not left behind.^[10–12,16]^

An ACL reconstruction in a skeletally young patient can cause valgus deformity.^[19]^ Treatment of osteochondral avulsion fractures of the ACL is focused on bony union rather than reconstruction of the ligament. Due to fragmentation, a suture repair was preferred over screw fixation. A transepiphyeal drill-hole of 2 mm in diameter (damaging 3% of the growth plate) did not cause a persistent development interruption in the distal femoral growth plate according to Mäkelä et al^[20]^ A drill hole of 3.2mm (damaging nearly 7% of the growth plate) caused permanent growth disruption and the shortening of the femur length. By using the transosseous tunnels inside-out technique described in this study, we observed that a very minor disruption of the growth plate of the distal femur condyle has occurred.

Langenhan et al^[11]^ described the technique of arthroscopic reduction and internal fixation for the femoral avulsion fracture of the ACL in a 14-year-old girl treated using K-wires. After 3 months, the K-wire was detached, and there was no disruption of the growth plate was seen. A bone plug or hardware across the growth plates, according to Koman and Sanders,^[21]^ is suggested to be contraindicated and should be refrained till the skeletal maturity is attained. We, therefore, use No. 2Ethibond sutures to reattach the avulsed fragment to its normal anatomical position and secure it with a proximal tie. There was no disruption of the growth plate reported 24 months following surgery.

The incidence of midsubstance rips in young people has been reported to increase as young people advance from young adulthood to puberty. Despite osteochondral avulsions from the ACL’s femoral attachment point being infrequent, doctors should be aware in this context too to ensure that the cases of such type should be considered and treated as soon as possible. Osteochondral avulsion fractures of the anterior cruciate ligament are treated by hard association rather than by creating a new tendon. Therefore, early diagnosis and treatment are vital, as postponing could cause malunion. Additionally, failure in the assessment of ACL-deficient knee will almost certainly result in knee shakiness, which, according to Millett et al,^[22]^ increases the likelihood of related knee injuries.

## 4. Conclusions

In conclusion, in the present case study, the use of the arthroscopic reduction technique and internal fixation by using sutures in an inside-out fashion is a precise and successful treatment strategy for treating fractures of ACL avulsion, which could assist in preventing persistent ligamentous laxity and extremely durable development aggravation.

## Author contributions

All authors have made substantial contributions to the conception of this study. All authors approved the final manuscript as submitted and agree to be accountable for all aspects of the work.

Conceptualization: Lei Wang, Longfei Ma.

Data curation: Lei Wang, Ke Tian.

Investigation: Zhongren Zheng

Writing—original draft: Zhongren Zheng, Longfei Ma.

Writing—review & editing: Xiaowei Zhao, Lei Wang, Longfei Ma.

The patient provided informed consent for the publication of this case.

## References

[1] Andrish JT. Anterior cruciate ligament injuries in the skeletally immature patient. Am J Orthop (Belle Mead NJ). 2001;30:103–10. 11234936

[2] Bradley GW Shives TC Samuelson KM. Ligament injuries in the knees of children. J Bone Joint Surg Am. 1979;61:588–91. 438247

[3] Clanton TO DeLee JC Sanders B. Knee ligament injuries in children. J Bone Joint Surg Am. 1979;61:1195–201. 511880

[4] Meyers MH McKeever FM. Fracture of the intercondylar eminence of the tibia. J Bone Joint Surg Am. 1970;52:1677–84. 5483091

[5] Bengtson H Giangarra C. Osteochondral avulsion fracture of the anterior cruciate ligament femoral origin in a 10-year-old child: a case report. J Athl Train. 2011;46:451–5. 21944079 10.4085/1062-6050-46.4.451 PMC3419159

[6] Corso SJ Whipple TL. Avulsion of the femoral attachment of the ACL in a 3-year-old boy. Arthroscopy. 1996;12:95–8. 8838737 10.1016/s0749-8063(96)90227-3

[7] Eady JL Cardenas CD Sopa D. Avulsion of the femoral attachment of the anterior cruciate ligament in a seven-year-old child. A case report. J Bone Joint Surg Am. 1982;64:1376–8. 7142249

[8] Edwards MR Terry J Gibbs J. Proximal anterior cruciate ligament avulsion fracture in a skeletally immature athlete: a case report and method of physeal sparing repair. Knee Surg Sports Traumatol Arthrosc. 2007;15:150–2. 16937153 10.1007/s00167-006-0154-2

[9] Kawate K Fujisawa Y Yajima H. Avulsion of the cartilaginous femoral origin of the anterior cruciate ligament in a three-year-old child. A case report with a thirteen-year follow-up. J Bone Joint Surg Am. 2004;86:1787–92. 15292430 10.2106/00004623-200408000-00026

[10] Lakshmanan P Sharma A Dixit V. Avulsion of the anterior cruciate ligament from femoral condyle: an unusual case report and a review of the literature. Knee Surg Sports Traumatol Arthrosc. 2006;14:1176–9. 16715316 10.1007/s00167-006-0090-1

[11] Langenhan R Baumann M Hohendorff B. Arthroscopically assisted reduction and internal fixation of a femoral anterior cruciate ligament osteochondral avulsion fracture in a 14-year-old girl via transphyseal inside-out technique. Strategies Trauma Limb Reconstr. 2013;8:193–7. 24002803 10.1007/s11751-013-0175-6 PMC3800520

[12] Pai SK Aslam Pervez N Radcliffe G. Osteochondral avulsion fracture of the femoral origin of the anterior cruciate ligament in an 11-year-old child. Case Rep Med. 2012;2012:506798. 22666266 10.1155/2012/506798 PMC3362875

[13] Robinson SC Driscoll SE. Simultaneous osteochondral avulsion of the femoral and tibial insertions of the anterior cruciate ligament. Report of a case in a thirteen-year-old boy. J Bone Joint Surg Am. 1981;63:1342–3. 7287808

[14] Tohyama H Kutsumi K Yasuda K. Avulsion fracture at the femoral attachment of the anterior cruciate ligament after intercondylar eminence fracture of the tibia. Am J Sports Med. 2002;30:279–82. 11912101 10.1177/03635465020300022201

[15] Uhorchak JM White PM III Scully TJ. Type III-A tibial fracture associated with simultaneous anterior cruciate ligament avulsion from the femoral origin. Am J Sports Med. 1993;21:758–61. 8238724 10.1177/036354659302100525

[16] Wardle NS Haddad FS. Proximal anterior cruciate ligament avulsion treated with TightRope® fixation device. Ann R Coll Surg Engl. 2012;94:e96–8. 22391373 10.1308/003588412X13171221589216 PMC5827257

[17] Wasilewski SA Frankl U. Osteochondral avulsion fracture of femoral insertion of the anterior cruciate ligament. Case report and review of literature. Am J Sports Med. 1992;20:224–6. 1558255 10.1177/036354659202000224

[18] Mizuta H Kubota K Shiraishi M. The conservative treatment of complete tears of the anterior cruciate ligament in skeletally immature patients. J Bone Joint Surg Br. 1995;77:890–4. 7593101

[19] Kocher MS Saxon HS Hovis WD. Management and complications of anterior cruciate ligament injuries in skeletally immature patients: survey of the herodicus society and the ACL study group. J Pediatr Orthop. 2002;22:452–7. 12131440

[20] Mäkelä EA Vainionpää S Vihtonen K. The effect of trauma to the lower femoral epiphyseal plate. An experimental study in rabbits. J Bone Joint Surg Br. 1988;70:187–91. 3346285 10.1302/0301-620X.70B2.3346285

[21] Koman JD Sanders JO. Valgus deformity after reconstruction of the anterior cruciate ligament in a skeletally immature patient. A case report. J Bone Joint Surg Am. 1999;81:711–5. 10360701 10.2106/00004623-199905000-00014

[22] Millett PJ Willis AA Warren RF. Associated injuries in pediatric and adolescent anterior cruciate ligament tears: does a delay in treatment increase the risk of a meniscal tear? Arthroscopy. 2002;18:955–9. 12426537 10.1053/jars.2002.36114

